# Wild canids as sentinels of ecological health: a conservation medicine perspective

**DOI:** 10.1186/1756-3305-2-S1-S7

**Published:** 2009-03-26

**Authors:** A Alonso Aguirre

**Affiliations:** 1 Wildlife Trust, 460 West 34th St., 17th Fl, New York, NY 10001, USA

## Abstract

The extinction of species across the globe is accelerating, directly or indirectly due to human activities. Biological impoverishment, habitat fragmentation, climate change, increasing toxification, and the rapid global movement of people and other living organisms have worked synergistically to diminish ecosystem function. This has resulted in unprecedented levels of disease emergence, driven by human-induced environmental degradation, which poses a threat to the survival and health of biodiversity. The emerging discipline of conservation medicine addresses these concerns through the following entities: humans; global climate; habitat destruction and alteration; biodiversity, including wildlife populations; domestic animals; and pathogens, parasites and pollutants. Furthermore, conservation medicine focuses on explicit linkages between these entities. As a crisis discipline, the usefulness of conservation medicine ultimately will depend on its applicability to solving problems. The perspectives and scientific findings of conservation medicine provide input into biomedical education; and policy and management of ecosystems, habitats and imperiled species. A sentinel species is one that has presented itself, or has been selected, to provide insight into the state (health) of an ecosystem, based on user-defined (e.g., researchers, conservationists or policymakers) objectives (e.g., disease, parasites, toxics, climate change, habitat destruction), coupled with the utility and vulnerability of this species to the perceived stress. The scientific information generated by the sentinel species should empower stakeholders and decision-makers to take mitigative action or support predictive capabilities; the "utility" of the species selected should consider its value and relevance to conservationists and to society at large (e.g., education and outreach; social sciences). Wild canids may serve as excellent sentinel species of emerging canine vector-borne diseases. Several canine vector-borne diseases or antibodies to these pathogens have been identified in wild canids including visceral leishmaniosis, Lyme disease, heartworm, hepatozoonosis and anaplasmosis to name a few. These reports are relatively recent as they relate to wildlife-domestic animal interactions, globalisation, translocations, habitat fragmentation and climate change. These pathogens and their relationship to wild canids are described herein. Further research needs to be performed to elucidate the role of the 36 extant species of wild canids in the epidemiology of canine vector-borne diseases.

## Background

The global loss of biological diversity affects the well being of both animals and people. Human impact on ecosystems and ecological processes is well documented. Habitat destruction and species loss have led to ecosystem disruptions that include, among other impacts, the alteration of disease transmission patterns (i.e., emerging diseases), the accumulation of toxic pollutants and the invasion of alien species and pathogens [1]. Ecological perturbations are creating a medium for new disease patterns and health manifestations. For example, in the marine environment, new variants of *Vibrio cholerae *have been identified within red tide algal blooms. These toxic blooms are occurring in greater frequency and size throughout the temperate coastal zones of the world. In arid zones of the southwestern United States, Brazil and Argentina, hantavirus epidemics have emerged in ecosystems that exhibit habitat degradation and climatic disturbances [2]. These brief examples illustrate our growing awareness of the interrelationship between health and the environment. When the natural resilience of ecosystems is stressed and barriers to disease transmission are reduced the emergence, resurgence and redistribution of infectious diseases are obvious symptoms of a deteriorating planet. According to the World Health Organization [3], 30 new diseases have been described in people including AIDS, Legionnaire's disease, and toxic *E. coli *infections since the mid 1970s. Diseases like tuberculosis, temperate-zone malaria, hemorrhagic dengue fever and diphtheria are also re-emerging as threats.

Anthropogenic change can be considered the primary factor causing the emergence of infectious diseases including vector-borne diseases. Global warming, human population growth, deforestation, globalisation, wildlife trade and pollution of oceans and freshwater bodies may have an impact on the prevalence and distribution of infectious pathogens. The dynamics of disease emergence from wildlife are complex and bring human and domestic animal populations into increasing contact with wild animals potentially infecting wildlife with new pathogens causing high mortality, decline and even local extinctions. In some cases, wildlife will survive infection and will become reservoirs. As human populations continue to augment exponentially and globalisation is imminent with increased travel and trade, these anthropogenic pressures on wildlife habitat and populations also will increase. The result can be predicted as a continuing spillover of new pathogens shared among wildlife, domestic animals and humans [4,5].

## Conservation medicine: ecological health in practice

Conservation medicine studies the two-way interactions between pathogens and disease on the one hand, and species and ecosystems on the other. It focuses on the study of the ecological context of health and the remediation of ecological health problems. In response to the growing health implications of environmental degradation, conservation medicine purview includes examining the linkages among a) changes in habitat structure and land use; b) emergence and re-emergence of infectious agents, parasites and environmental contaminants; c) maintenance of biodiversity and ecosystem functions as they sustain the health of plant and animal communities including humans. Conservation medicine is concerned with the effects of disease on rare or endangered species and on the functioning of ecosystems. It is also concerned with the impacts of changes in species diversity or rarity on disease maintenance and transmission. The dynamic balance that we term "health" is viewed on a series of widely varying spatial scales by many disciplines including human and public health, epidemiology, veterinary medicine, toxicology, ecology and conservation biology. Conservation medicine represents an approach that bridges these disciplines to examine health of individuals, groups of individuals, populations, communities, ecosystems and the landscapes in which they live as an indivisible continuum, it is truly a transdiscipline. By reaching out to multiple disciplines, conservation medicine provides new skills, tools and vision to the field of both conservation biology and medicine. This includes bringing biomedical research and diagnostic resources to address conservation problems, e.g., development of new non-invasive health monitoring techniques; training veterinarians, physicians and conservation biologists in the promotion and practice of ecological health; and by establishing trans-disciplinary teams of health and ecological professionals to assess and address ecological health problems [4-7].

The ecological impacts of humans can ripple throughout ecological communities. The demise of one species, or the rise of one species at the expense of another may establish a trophic cascade of ecological responses. When predator-prey or species competition relationships are disrupted, ecological impacts may extend beyond the predator and prey or the competitors [8-10]. The spread of Lyme disease as a result of the changing ecology of white-tailed deer (*Odocoileus virginianus*) and white-footed mice (*Peromyscus leucopus*) in a landscape devoid of large predators and diminished biodiversity is a good example of ecological magnification of disease. Until recently we are beginning to understand the "dilution effect" as empirical evidence that host diversity decreases infection and transmission of infectious diseases [11].

Conservation medicine receives input from both the social sciences and bioinformatics. Fields such as sociology, economics and anthropology inform the science and the practice of conservation medicine by revealing potential causes of human behaviour relevant to human-induced environmental change. Top-down approaches to wildlife management are being replaced by adaptive management strategies that reflect uncertainty and complexity. These new management techniques are necessary to further develop the field. Bioinformatics and the creation, management and dissemination of databases relevant to wildlife, their habitats and their diseases may be crucial to this new discipline.

### Global climate change and disease emergence

Climate change is one of the most pressing ecological health concerns today. By all scientific evidence climate change is a reality. The health consequences are pervasive. The effects of climate change in promoting the spread of infectious disease from more tropical ranges to temperate areas are being observed in such diseases as malaria and dengue fever [2,9,12]. The impact of climate change on all ecological processes is profound: increased precipitation in some regions and drought in others; increased erosion of the coastal zone with rising sea levels; and the inability of many species to adapt to the relatively rapid changes in climatic regimes potentially resulting in mass extinctions [13,14]. Changes in atmospheric volatilisation and deposition of pollutants are of equal concern. The impacts of climate change on biodiversity are still underestimated. Up to 37% of over 1,000 animal and plant species in six regions of the planet are at risk of extinction from climate change. Several scenarios produce projections of species committed to extinction of 18% (minimal climate-warming), 24% (mid-range warming) and 35% (maximum-warming) [15,16]. A more recent example described by Rausch *et al. *[17] hypothesized that retreating sea ice is changing the feeding behavior of pagophilic pinnipeds such as the walrus (*Odobenus rosmarus*) with a usual diet of benthic invertebrates inhabiting coastal waters to a depth of approximately 100 m now shifting to a more pelagic diet including ring seals (*Pusa hispida*) due to the retreating ice substrate, thus inducing to modification of the helminth fauna. A higher prevalence of trichinellosis caused by *Trichinella spiralis *has been observed with a huge zoonotic potential to indigenous peoples feeding on these pinnipeds. Trichinellosis is widespread worldwide with a generally low prevalence and restricted to specific areas in Europe. Low intensity infections occur in wildlife (wild boar, red fox, raccoon dog, polar bear, rodents), keeping alive the risk of infection to humans [18].

Current evidence suggests that inter-annual and inter-decadal climate variability have a direct influence on the epidemiology of vector-borne diseases. Opportunists that multiply rapidly such as insects, rodents and microorganisms colonize stressed environments at higher rates.

Because they are sensitive to climate, the distribution and number of vectors are also affected by climate change. According to the IPCC 2007 Climate Change Report , climate change has already altered the distribution of some disease vectors. There is evidence that the geographic range of ticks and mosquitoes that carry disease has changed in response to climate change. Tick and mosquito vectors have extended their range North in Sweden and Canada and into higher altitudes in other countries worldwide. While future climate change is expected to continue to alter the distribution of disease vectors, it is important to recognize that there are several other factors (such as changes in land use, population density and human behavior) that can also change the distribution of disease vectors as well as the extent of infection.

Climate is closely associated with the natural history of vectors so warmer temperatures will increase their range and distribution including altitudinal gradients. *Ixodes pacificus *is the main tick vector for transmission of *Anaplasma phagocytophilum *and *Borrelia burgdorferi *to large vertebrates in California. A recent study examined spatial and temporal relationships among *A. phagocytophilum-*and *B. burgdorferi*-exposed coyotes (*Canis latrans*) with vegetation type and climate. The overall seroprevalences were 39.5% and 18.9%, respectively. Increased sero-prevalence was a positive function of rainfall [19].

### Vector-borne diseases: a threat to human health and conservation

The role of emerging vector-borne diseases on global health cannot be underestimated within the context of conservation medicine. Parasitic vector-borne diseases are classical emerging infectious diseases in human populations such as leishmaniosis, Lyme disease and ehrlichiosis among others. Eukaryotic parasites are among the most significant agents of emerging diseases of wildlife and domestic animals [20]. Parasites are part of biological diversity; however, pathogenic species may pose a threat to conservation of their natural hosts or of new species of hosts. A striking example cited is translocation of animals from their natural habitat to another habitat or to a different region of the world when their parasite fauna are also exposed to new species of hosts. In fact, when the new hosts become infected with these novel parasites, the pathogenicity and epidemiology of the infection become unpredictable. The introduction of a parasite to a new environment may or may not affect population density of the host, but runs the risk of introducing non-indigenous parasitic infections [18].

Vector-borne diseases of wildlife have implications for wildlife conservation, for evaluating wildlife-domestic interactions, and for determining their public health significance. Both parasite and host adapt to one another in a mutual co-existence [21]. When a population of free-ranging wildlife is exposed to an emerging infectious or parasitic disease the response of the host might be to (a) resist the parasite, (b) develop severe clinical parasitism or (c) adapt to the new parasite and become a carrier. The source of exposure may be from: (1) domestic animals, (2) re-introduction of wild animals (raised in captive propagation programmes) and/or (3) the spread of a related species in new habitats [18].

### Cross-species transmission

The importance of parasites in wild mammals and their association with domestic animals and humans cannot be overemphasised. Forrester [22] reported that 119 parasites and infectious diseases were interchangeable between wild and domestic mammals in Florida. From these, more than 46% were helminths (45 nematodes, five cestodes, four trematodes and one acanthocephalan) when wild and domestic swine were included. In addition, 10% (seven nematodes, four cestodes and one trematode) were considered zoonotic.

The movement of wild animals can also introduce exotic diseases into the native population and conversely, can expose translocated animals to diseases for which they have not developed resistance. Several species are susceptible to diseases of domestic animals and humans. The possibilities of transmission are potentially high during capture, transport and release. Emerging parasites in new hosts are becoming more evident as diagnostic techniques are refined, wildlife-human-domestic animal interactions increase and new diseases are identified [18].

The threat of disease transmission from domestic animals to wildlife has become recognised as an increasing concern within the conservation community in recent years. Domestic dogs pose a significant risk as reservoirs for infectious diseases, especially for wild canids. As human populations expand into wild habitats with their pets and livestock the greater the risk of emerging infections reaching rare species or vice versa. Population density represents a disease risk in these situations where feral dogs have established large packs. Dogs may exist in very high densities, so where infection was once sporadic it may turn potentially into an epidemic that in the right circumstances may spillover to wild populations [23]. A classical example is the devastating outbreak of canine distemper in Serengeti lions (*Panthera leo*). It was estimated that the disease killed over 1000 lions, a third of the Serengeti the population, before spreading North across the border in the Masai Mara National Reserve in Kenya by August 1994, with a neurologic syndrome. Over 85% of the surviving Serengeti lions were found with anti-CDV antibodies. Also, uncounted hyaenas, bat-eared foxes and leopards were also affected [24]. Speculation exists on how this outbreak was initiated, as village dogs rarely get sufficiently close to a lion to pass on the virus, and there are likely other reservoir hosts. Spotted hyaenas (*Crocuta crocuta*) are probably the final link in the chain, because they mix with lions at the kill [23-25]. Canine distemper may have played a part in the extinction of the marsupial wolf *Thylacinus *spp. in the early 20th century, and it pushed the black-footed ferret (*Mustela nigripes*) of North America to the brink of extinction in the 1970s [23,26]. During the early 1990s rabies claimed more than half of the Ethiopian wolves (*Canis simensis*) in the Bale Mountain National Park. Rabies was also responsible for the death of African wild dogs (*Lycaon pictus*) in the Masai Mara reserve in Kenya in 1989 and in the Serengeti in 1990 [27].

An assessment of which pathogens might pose a problem and their patterns of infection in natural hosts is necessary with increasing awareness of disease as an endangering process. The exposure of sympatric Ethiopian wolves and domestic dogs to canine distemper virus (CDV), canine adenovirus (CAV) and canine parvovirus (CPV) in the Bale region, Ethiopia, has been documented [28]. The wolf population in the region declined in the late 1980s and early 1990s, with rabies responsible for a dramatic population reduction between 1990 and 1992. Although the population declined further up until 1995, it is not possible to assess whether the concurrent canine distemper epidemic in park dogs also affected wolves. Nevertheless, with evidence of rabies, CDV, CAV and CPV infections in sympatric domestic dogs and Ethiopian wolves, canid diseases clearly pose a significant threat to the future persistence of this Ethiopian wolf population [28].

## Vector-borne diseases and wild canids

Canine vector-borne diseases are emerging and re-emerging following global patterns. For example, leishmaniosis in the Americas caused by *Leishmania braziliensis *has been highly correlated to deforestation and human penetration. Previously considered an exotic disease, canine leishmaniosis caused by *Leishmania infantum *has recently been detected within the foxhound population in the United States and parts of Canada. The following sections describe selected canine vector-borne diseases and their relationship to wild canid species as reported in the literature and their imminent emergence across continents. Diseases are described in alphabetical order for practical purposes.

### Anaplasmosis

*Anaplasma phagocytophilum *(formerly *Ehrlichia phagocytophila*, *Ehrlichia equi *and *Anaplasma phagocytophila*) is the causative agent of granulocytic ehrlichiosis (anaplasmosis) in humans, horses, sheep, cattle, dogs and cats. Over 450 European sheep ticks (*Ixodes ricinus*) collected from 100 red foxes (*Vulpes vulpes*) in Hungary were tested for the pathogen as 112 pools each containing five or fewer ticks from one fox. Six foxes were found infected in the PCR-based test [29]. The epidemiological consequences of these findings are unknown.

### Babesiosis

Young coyotes (*Canis latrans*) were experimentally infected with *Babesia gibsoni *developing pale mucous membranes, splenomegaly and a positive haeme reaction in urine. One of the coyotes exhibited mild depression and anorexia. The mild clinical signs coupled with the high level and long duration of parasitemia indicated that coyotes could serve as reservoirs of this emerging disease [30].

### Bartonellosis

*Bartonella vinsonii *subsp. *berkhoffii *was originally isolated from a dog with infectious endocarditis and was recently identified as a zoonotic agent causing human endocarditis. An epidemiological study was conducted following a child bitten by a coyote who developed clinical signs compatible with *Bartonella *infection in California. Among 109 coyotes from central coastal California, 31 animals (28%) were found to be bacteremic with *B. vinsonii *subsp. *berkhoffii *and 83 animals (76%) presented antibodies. These findings suggest these animals could be the wildlife reservoir. Further studies are necessary to elucidate the mode of transmission especially to identify potential vectors and to determine how humans become infected [31].

### Borreliosis

Different *Borrelia *species and serotypes were tested for their sensitivity to serum complement from various animals and humans; and wolves were identified as a probable reservoir in nature [32]. Seven of 100 skin samples of red foxes (*Vulpes vulpes*) were tested positive for *B. burgdorferi *in Brandenburg, Germany [33]. A new species denominated *Candidatus B. texasensis *was isolated in 1998 from an adult male *Dermacentor variabilis *tick feeding on a coyote from Texas, characterised with several molecular techniques, but isolation attempts were not fruitful [34]. Coyotes have been previously used as sentinels of Lyme disease to correlate with infection in humans in the USA. One thousand canine sera (917 dogs, 83 coyotes) obtained from field personnel were screened with an ELISA test and results were validated by Western blot and indirect fluorescent antibody (IFA) tests at reference laboratories. A total of 22/1000 canines were confirmed serologically positive (21 dogs and 1 coyote; seroprevalence 2.3% and 1.2%, respectively). The low prevalence of seropositivity in sentinel canines suggests the Lyme disease hazard is minimal in San Diego, California [35].

### Canine heartworm

Canine heartworm caused by *Dirofilaria immitis *affects wild canids and may be a factor impacting the health and population dynamics of coyotes. Coyotes may serve also as a potential reservoir for transmission of these parasites to domestic dogs. Almost a 1000 coyotes were surveyed in Illinois for the presence of heartworm identifying a statewide prevalence of 16%. The authors concluded that heartworm disease is only a minor factor influencing coyote population dynamics in Illinois and possesses perhaps low risk to the domestic canine population [36].

As part of a multifaceted ecologic study of maned wolves (*Chrysocyon brachyurus*) and other canids in the large, remote Noel Kempff Mercado National Park in northeastern Bolivia, 40 domestic dogs in two villages and at two smaller settlements bordering the national park were sampled for exposure to canine diseases. High levels of exposure were found to CDV and CPV, both of which are known to cause mortality in maned wolves and other carnivores. Moderate to high levels of exposure were found to rabies virus, *E. canis*, and *Toxoplasma gondii*, as well as significant levels of infection with *D. immitis*. This study reports evidence of exposure to several diseases in the domestic dogs bordering the park. Contact between wild carnivores and dogs has been documented in the sampled villages, therefore dogs likely pose a substantial risk to the carnivores within and near the park. Further measures should be undertaken to decrease the risk of spillover infection from domestic animals into the wild species of this region [37]. Maned wolves are neotropic canids, listed as a CITES Appendix II species, with a distribution in Argentina, Brazil, Bolivia and Paraguay. Primary threats to the survival of free-ranging wolves include habitat loss, road kills and shooting by farmers. An additional threat is the risk of morbidity and mortality due to infectious and parasitic diseases [37]. It has been documented that captive maned wolves are susceptible to, and die from, common infectious diseases of domestic dogs including CDV, CPV, rabies virus and CAV. A survey documenting these agents indicated that free-ranging wolves in the same park in Bolivia have been exposed to multiple infectious and parasitic agents of domestic carnivores, including the viruses previously mentioned, canine coronavirus, rabies virus, *Leptospira interrogans *spp., *T. gondii *and *D. immitis*, and may be at increased disease risk due to these pathogens originating in the domestic dog population [38].

### Canine Ehrlichiosis

A seroepidemiological survey was conducted to investigate the prevalence of antibodies reactive with the *Ehrlichia canis *and *E. phagocytophila *(newly named *Anaplasma phagocytophilum*) genogroup antigens, and the spotted-fever group rickettsiae antigens in jackals (*Canis aureus syriacus*) in Israel, to assess the possible role of this species in the epidemiology of these diseases. A total of 53 serum samples were tested and antibodies were detected in 36% to *E. canis*, 26% were positive to *E. chaffeensis*. In addition, 26% were seropositive to *E. phagocytophila *[39]. In another study performed in coyotes from California, a total of 68/149 (46%) samples were seropositive to *E. equi*, two (1%) to *E. risticii *(newly named *Neorickettsia risticii*) and none of the samples had antibodies reactive to *E. canis *demonstrating that coyotes have been exposed to granulocytic ehrlichiae and *E. risticii *and may play a role in the epidemiology of these ehrlichial agents in California [40]. Red foxes and grey foxes (*Urocyon cinereoargenteus*) were evaluated for their susceptibility to experimental infection with *E. chaffeensis*, the causative agent of human monocytotropic ehrlichiosis [41]. The results imply that red foxes, but not grey foxes, are potential vertebrate reservoirs illustrating the need to verify serologic evidence of *E. chaffeensis *infection among wild animals.

### Canine visceral leishmaniosis (CVL)

CVL has been recently documented in wild canids in endemic regions of Europe. Pathologic and parasitologic analysis of a grey wolf (*Canis lupus*) found dead and confirmed by PCR, indicated that lesions associated with infection by *L. infantum *were typical for CVL commonly described in dogs [42]. A serologic survey of captive wolves in southwestern Europe determined that three of 33 wolves (9%) presented low levels of antibodies to *L. infantum *infection using an ELISA test [43]. An epizootiological survey of leishmaniosis in 67 foxes (*Vulpes vulpes*) was conducted in Guadalajara, Spain, identifying a prevalence of 74% positives determined by molecular methods [44].

Visceral leishmaniosis occurs in many parts of the world and dogs are considered a major reservoir host for human infections. Human visceral leishmaniosis has recently emerged as an opportunistic infection among individuals co-infected with HIV/AIDS and in persons taking immunosuppressive drugs. Royspal *et al. *[45] reported 3 naturally infected foxhounds from Virginia by culture. The disease has been documented extensively in Brazil. Among 15 captive canids from a zoo in Belo Horizonte, Minas Gerais, Brazil, two animals, a bush dog (*Spheotos venaticos*) and a hoary fox (*Lycalopex vetulus*), were serologically positive and developed clinical signs of CVL, whereas three other canids, including a crab-eating fox (*Cerdocyon thous*), a maned wolf and a hoary fox had positive serological results without clinical signs [46]. During 1999–2003 parasitology and serology tests were performed in domestic (n = 1568) and wild jackals (*Canis aureus*, n = 10), red foxes (n = 10) and wolves (n = 10) in Iran demonstrating that 10% of wild canids were infected by *L. infantum*. Ten out of 11 *Leishmania *spp. isolated from dogs and wild canines were identified as *L. infantum *and one other as *L. tropica *by molecular and biochemical techniques [47].

### Hepatozoonosis

The first PCR detection of *Hepatozoon canis *in a naturally infected red fox from Slovakia, a *Rhipicephalus sanguineus*-free region, was recently performed [48]. It is believed that the infection was spread by infected *R. sanguineus *that might have been brought to Slovakia by travellers, golden jackals, or foxes migrating because of expansion of golden jackals and environmental and climate changes. A seroepidemiological survey was conducted to investigate the prevalence of antibodies reactive with *E. canis *and *H. canis *antigens in free-ranging red foxes in Israel. Of 84 fox sera assayed, 36% were seropositive for *E. canis *by IFA test and 24% were positive using the ELISA test. Canine ehrlichiosis and hepatozoonosis appear to be endemic in the wild red fox populations in Israel, and foxes may serve as a reservoir for infection of domestic dogs and other wild canine species [49].

## Wild canids as sentinels of ecological health

The canidae family includes 16 recent genera distributed in most land masses of the world. Nine of the 36 canid taxa are threatened: Darwin's fox, island fox and red wolf are listed as Critically Endangered, while Ethiopian wolf, African wild dog and dhole (*Cuon aplinus*) are endangered. Others are rare and even declining, while many wild canids like coyotes are common, therefore they are involved in major wildlife management issues (such as disease transmission, predation on livestock, sport hunting, fur trade). Many wild canid species may serve as sentinels of ecological health as they may be critical to proactively identify potentially pathogenic canine vector-borne diseases emerging parasites.

Sentinel species are the proverbial "canaries in the mine shaft". They serve as indicators of their environment and may reflect the quality of health in their ecosystems. The scientific information generated by the sentinel species should empower stakeholders to take mitigative action or support predictive capabilities. The "utility" of the species selected should consider its value and relevance to decision makers, conservationists and to society at large. Assessing the health an ecosystem will require of a "suite" of sentinel species including different trophic levels, ecological roles, taxa and different spatial/temporal scales. It will require conservation medicine teams to apply transdisciplinary efforts. It will require the involvement of decision makers and the general public to change our behaviour towards the planet [50,51].

Sentinel species may assist in increasing monitoring efficiency at the ecosystem level. They can be utilised during rapid risk assessments to provide information on the environmental conditions of an area during an emerging parasitic disease outbreak. Sentinel species or health indicator species can be selected for their ability to reflect environmental perturbations. Based on their life history and physiological attributes, selected species can provide insightful information about environmental changes at various spatial, temporal and trophic scales. Given the complexity of ecosystems, sentinel species should be thought of as being specific to particular environmental conditions during a pandemic. In some cases, an assemblage of species may be suitable for providing a 'umbrella' effect in monitoring the cumulative impacts of multiple environmental variables that create the complexity of an emerging canine vector-borne disease [50,51].

On the one hand, wild canid populations may suffer severe epizootics and declines related to domestic dog diseases; on the other hand, there are species like jackals, coyotes and foxes that are highly adaptable to ecosystems and human-impacted environments and are reservoirs of several diseases of zoonotic or domestic animal importance including rabies, parvovirus infection, canine distemper and mange. These species are ideal to monitor vector-borne diseases. In addition, wild caninds adapted to the human condition host innumerable parasites, bacteria and viruses, which in turn may contribute to density-dependent mortality. By increasing wildlife-domestic animal interactions the potential of spread of disease to domestic dog populations is real [25,52]. Further research is necessary to identify wild reservoirs, susceptible hosts and zoonotic interactions for all canine vector-borne diseases at a global level and to establish protective measures to dwindling wild canid populations such as wolves to ensure their long-term survival.

## Competing interests

The author declares that they have no competing interests.

## References

[B1] Aguirre AA, Tabor GM (2008). Global factors driving emerging infectious diseases: Impact on wildlife populations. Animal Biodiversity and Emerging Diseases: Annals of the New York Academy of Sciences.

[B2] Epstein PR (1999). Climate and health. Science.

[B3] World Health Organization (1996). The world health report: fighting disease, fostering development.

[B4] Aguirre AA, Eds (2002). Conservation medicine: ecological health in practice.

[B5] Tabor GM, Ostfeld RS, Poss M, Dobson AP, Aguirre AA, Soulé ME, Orians GH (2001). Conservation biology and the health sciences: defining the research priorities of conservation medicine. Research Priorities in Conservation Biology.

[B6] Meffe G (1999). Conservation medicine. Conservation Biology.

[B7] Pokras M, Tabor GM, Pearl M, Sherman D, Epstein P, Raven P, Williams T (1999). Conservation medicine: an emerging field. Nature and Human Society: the quest for a sustainable world.

[B8] Estes JA, Tinker MT, Williams TM, Doak DF (1998). Killer whale predation on sea otters linking oceanic and nearshore ecosystems. Science.

[B9] Epstein PR, Dobson A, Vandermeer J, Grifo F, Rosenthal J (1997). Biodiversity and emerging infectious diseases: integrating health and ecosystem monitoring. Biodiversity and Human Health.

[B10] Pace ML, Cole JJ, Carpenter SR, Kitchell JF (1999). Trophic cascades revealed in diverse ecosystems. TREE.

[B11] Ostfeld RS, Keesing F (2000). Biodiversity and disease risk: the case of Lyme disease. Conservation Biology.

[B12] Patz JA, Epstein PR, Burke TA, Balbus JM (1996). Global climate change and emerging infectious diseases. JAMA.

[B13] McMichael AJ (1997). Global environmental change and human health: impact assessment, population vulnerability, and research priorities. Ecosystem Health.

[B14] McMichael AJ, Bolin B, Costanza R, Daily GC, Folke C, Lindahl-Kiessling K, Lindgren B, Niklasson E (1999). Globalization and the sustainability of human health: an ecological perspective. Bioscience.

[B15] Root TL, Price JT, Hall KR, Schneider SH, Rosenzweig C, Pounds JA (2003). Fingerprints of global warming on wild animals and plants. Nature.

[B16] Thomas CD, Cameron A, Green RE, Bakkenes M, Beaumont LJ, Collingham YC, Erasmus BFN, Ferreira de Siqueira M, Grainger A, Hannah L (2004). Extinction risk from climate change. Nature.

[B17] Rausch RL, George JC, Brower HK (2007). Effect of climatic warming on the Pacific walrus, and potential modification of its helminth fauna. J Parasitol.

[B18] Chowdhury N, Aguirre AA (2001). Helminths of wildlife: a global perspective.

[B19] Foley JE, Queen EV, Sacks B, Foley P (2005). GIS-facilitated spatial epidemiology of tick-borne diseases in coyotes (*Canis latrans*) in northern and coastal California. Comparative Immunol Microbiol Infect Diseases.

[B20] Cunningham AA, Daszak P, Rodriguez JP (2003). Pathogen pollution: defining a parasitological threat to biodiversity conservation. J Parasitol.

[B21] Aguirre AA, Starkey EE, Hansen DE (1995). Wildlife diseases in national park ecosystems. Wildl Soc Bull.

[B22] Forrester DJ (1992). Parasites and diseases of wild mammals in Florida.

[B23] Pain S (1997). The plague dogs. New Science.

[B24] Roelke-Parker ME, Munson L, Packer C, Kock R, Cleaveland S, Carpenter M, O'Brien SJ, Pospischil A, Hofmann-Lehmann R, Lutz H (1996). A canine distemper virus epidemic in Serengeti lions (*Panthera leo*). Nature.

[B25] Brickner I (2002). The impact of domestic dogs (*Canis familiaris*) on wildlife welfare and conservation. Final paper submitted to Prof Yoram Yom-Tov, Israel Unpublised report.

[B26] Williams ES, Thorne ET, Appel MJG, Belitsky DW (1998). Canine distemper in black-footed ferrets (*Mustela nigripes*) from Wyoming. J Wildl Diseases.

[B27] Kat PW, Alexander KA, Smith JS, Richardson JD, Munson L (1996). Rabies among African wild dogs (*Lycaon pictus*) in the Masai Mara, Kenya. J Vet Diagn Invest.

[B28] Laurenson K, Sillero-Zubiri C, Thompson H, Shiferaw F, Thirgood S, Malcolm J (1998). Disease as a threat to endangered species: Ethiopian wolves, domestic dogs and canine pathogens. Animal Conservation.

[B29] Sréter T, Sréterné Lancz Z, Széll Z, Egyed L (2004). *Anaplasma phagocytophilum*: an emerging tick-borne pathogen in Hungary and central eastern Europe. Ann Trop Med Parasitol.

[B30] Evers HV, Kocan AA, Reichard MV, Meinkot JH (2003). Experimental *Babesia gibsoni *infection in coyotes (*Canis latrans*). J Wildl Diseases.

[B31] Chang CC, Kasten RW, Chomel BB, Simpson DC, Hew CM, Kordick DL, Heller R, Piemont Y, Breitschwerdt EB (2000). Coyotes (*Canis latrans*) as the reservoir for a human pathogenic *Bartonella *sp.: molecular epidemiology of *Bartonella vinsonii *subsp. *berkhoffii *infection in coyotes from central coastal California. J Clin Microbiol.

[B32] Bhide MR, Travnicek M, Levkutova M, Culik J, Revajova V, Levkut M (2005). Sensitivity of *Borrelia *genospecies to serum complement from different animals and human: a host-pathogen relationship. Fems Immunology and Medical Microbiology.

[B33] Heidrich J, Schonberg A, Steuber S (1999). Investigation of skin samples from red foxes (*Vulpes vulpes*) in eastern Brandenburg (Germany) for the detection of *Borrelia burgdorferi *s. l. Zbl Bakt-Int J Med Microbiol.

[B34] Lin T, Gao LH, Seyfang A, Oliver JH (2005). '*Candidatus Borrelia texasensis'*, from the American dog tick Dermacentor variabilis. Int J Syst Evolut Microbiol.

[B35] Olson PE, Kallen AJ, Bjorneby JM, Creek JG (2000). Canines as sentinels for Lyme disease in San Diego County, California. J Vet Diagn Invest.

[B36] Nelson TA, Gregory DG, Laursen JR (2003). Canine heartworms in coyotes in Illinois. J Wildl Diseases.

[B37] Bronson E, Emmons LH, Murray S, Dubovi EJ, Deem SL (2008). Serosurvey of pathogens in domestic dogs on the border of Noel Kempff Mercado National Park, Bolivia. J Zoo Wildl Med.

[B38] Deem SL, Emmons LH (2005). Exposure of free-ranging maned wolves (*Chrysocyon brachyurus*) to infectious and parasitic disease agents in the Noel Kempff Mercado National Park, Bolivia. J Zoo Wildl Med.

[B39] Waner T, Baneth G, Strenger C, Keysary A, King R, Harrus S (1999). Antibodies reactive with *Ehrlichia canis*, *Ehrlichia phagocytophila *genogroup antigens and the spotted fever group rickettsial antigens, in free-ranging jackals (*Canis aureus syriacus*) from Israel. Vet Parasitol.

[B40] Pusterla N, Chang CC, Chomel BB, Chae JS, Foley JE, DeRock E, Kramer VL, Lutz H, Madigan JE (2000). Serologic and molecular evidence of *Ehrlichia *spp. in coyotes in California. J Wildl Diseases.

[B41] Davidson WR, Lockhart JM, Stallknecht DE, Howerth EW (1999). Susceptibility of red and gray foxes to infection by *Ehrlichia chaffeensis*. J Wildl Diseases.

[B42] Beck A, Beck R, Kusak J, Gudan A, Martinkovic F, Artukovic B, Hohsteter M, Huber D, Marinculic A, Grabar Z (2008). A case of visceral leishmaniosis in a gray wolf (*Canis lupus*) from Croatia. J Wildl Diseases.

[B43] Sastre N, Francino O, Ramirez O, Ensenat C, Sanchez, Altet L (2008). Detection of *Leishmania infantum *in captive wolves from southwestern Europe. Veterinary Parasitology.

[B44] Criado-Fornelio A, Gutierrez-Garcia L, Rodriguez-Caabeiro F, Reus-Garcia E, Roldan-Soriano MA, Diaz-Sanchez MA (2000). A parasitological survey of wild red foxes (*Vulpes vulpes*) from the province of Guadalajara, Spain. Vet Parasitol.

[B45] Rosypal AC, Troy GC, Zajac AM, Duncan RB, Waki K, Chang KP, Lindsay DS (2003). Emergence of zoonotic canine leishmaniasis in the United States: isolation and immunohistochemical detection of Leishmania infantum from foxhounds from Virginia. J Eukaryot Microbiol.

[B46] Luppi MM, Malta MCC, Silva TMA, Silva FL, Motta ROC, Miranda I, Ecco R, Santos RL (2008). Visceral leishmaniasis in captive wild canids in Brazil. Veterinary Parasitol.

[B47] Mohebali M, Hajjaran H, Hamzavib Y, Mobedia I, Arshic S, Zareia Z, Akhoundia B, Manouchehri Naeinid K, Avizehe R, Fakhara M (2005). Epidemiological aspects of canine visceral leishmaniosis in the Islamic Republic of Iran. Veterinary Parasitol.

[B48] Majláthová V, Hurniková Z, Majláth I, Pe ko B (2007). *Hepatozoon canis *infection in Slovakia: Imported or autochthonous?. Vector-Borne and Zoonotic Diseases.

[B49] Fishman Z, Gonen L, Harrus S, Strauss-Ayali D, King R, Baneth G (2004). A serosurvey of *Hepatozoon canis *and *Ehrlichia canis *antibodies in wild red foxes (*Vulpes vulpes*) from Israel. Veterinary Parasitology.

[B50] Aguirre AA, Tabor GM (2004). Marine vertebrates as sentinels of marine ecosystem health. EcoHealth.

[B51] Tabor GM, Aguirre AA (2004). Ecosystem health and sentinel species: adding an ecological element to the proverbial 'canary in the mineshaft'. EcoHealth.

[B52] Aguirre AA, Lefèvre P-C, Blancou J, Chermette R, Uilenberg G Parasitic diseases in wildlife and domestic animals: new trends of disease emergence. Infectious and Parasitic Diseases of Livestock.

